# Electrical Risk Score as a Predictor of Coronary Artery Disease

**DOI:** 10.3390/jcm14228106

**Published:** 2025-11-16

**Authors:** Özge Turgay Yıldırım, Tuğba Dişikırık, Gamze Yeter Arslan, Mehmet Semih Belpınar, Ayberk Beral, Barış Özden, Mehmet Özgeyik

**Affiliations:** 1 Eskişehir City Health Application and Research Center, Health Sciences University, 26080 Eskişehir, Turkey; 2 Kepez State Hospital, 07230 Antalya, Turkey; 3 Sivas Numune Hospital, 58000 Sivas, Turkey; mehmetsemihbelpinar@gmail.com

**Keywords:** electrical risk score, coronary artery disease, electrocardiography, prediction

## Abstract

**Background/Objectives:** Coronary artery disease (CAD) is a leading cause of global mortality, necessitating effective risk stratification tools for optimal patient management. The electrical risk score (ERS) is a multi-parametric index incorporating various electrocardiographic (ECG) parameters, previously shown to predict unfavorable cardiovascular outcomes. However, the relationship between ERS and the presence and severity of CAD remains unclear. This study aimed to investigate the association of ERS with the presence and extent of CAD as assessed by coronary angiography. **Methods:** This retrospective study included 314 consecutive patients who underwent coronary angiography. ERS was calculated using six ECG parameters: heart rate > 75 bpm, left ventricular hypertrophy, delayed QRS transition zone, frontal QRS-T angle > 90°, prolonged QTc interval, and extended T peak to T end interval. **Results:** Of the study population (mean age 57.8 ± 11.4, 61.5% male), 158 were diagnosed with CAD, and 156 constituted the control group. The mean ERS was significantly higher in the CAD group than the control group (2.34 ± 1.35 vs. 1.78 ± 1.12, *p* = 0.006). Among ERS components, delayed QRS transition (*p* = 0.023), prolonged QTc (*p* = 0.004), and extended T peak to T end interval (*p* = 0.001) were notably more prevalent in the CAD group. ERS was independently associated with the presence of CAD on multivariate logistic regression analysis (*p* < 0.05). **Conclusions:** ERS is significantly associated with the presence and severity of CAD in stable patients. Elevated ERS, particularly due to delayed QRS transition, prolonged QTc, and extended T peak to T end interval, may serve as a valuable, non-invasive marker for prediction and early identification of CAD.

## 1. Introduction

Coronary artery disease (CAD) remains the foremost cause of mortality worldwide, representing a major public health challenge [1]. Patients with CAD often present with a heterogeneous clinical spectrum, particularly during their initial evaluation [2]. Timely and accurate diagnosis followed by appropriate therapeutic interventions at the first medical contact are critical determinants of patient prognosis and long-term outcomes [1].

Electrocardiography (ECG) constitutes an essential, non-invasive, and widely accessible tool utilized in both the diagnosis and monitoring of CAD [3]. Beyond classical ECG markers, multiple depolarization and repolarization parameters may capture subclinical electrical remodeling related to ischemia, autonomic tone, ventricular hypertrophy, and dispersion of repolarization. While certain ECG changes allow rapid diagnosis in acute presentations without necessitating additional testing, the interpretation of ECG findings in non-acute or stable phases is more complex, often yielding limited diagnostic clarity [4].

To address this limitation in the stable phase, composite ECG-based indices that integrate multiple parameters have been explored to improve diagnostic and prognostic assessment. The electrical risk score (ERS) is one such multi-parametric index, incorporating heart rate, left ventricular hypertrophy, QRS transition zone, corrected QT interval, T peak to T end interval, and the frontal QRS-T angle [5]. ERS has shown promise in prognosticating adverse cardiovascular events across a variety of clinical scenarios, including acute coronary syndromes and sudden cardiac death [6,7,8].

The relationship between ERS and the presence and extent of coronary atherosclerosis, however, remains underexplored. Accordingly, this study focuses on patients with stable CAD and evaluates the association between ERS and the presence and severity of coronary artery disease as assessed by coronary angiography. Our objective is to clarify whether ERS may serve as a non-invasive indicator associated with angiographic CAD in the stable setting.

## 2. Materials and Methods

### 2.1. Patient Selection

**Figure 1:**
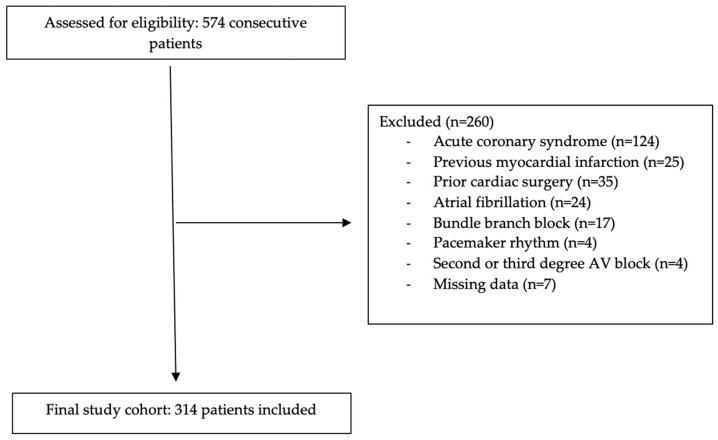
Flow chart of the study population.

A total of 574 consecutive patients over the age of 18 who underwent coronary angiography between January 2024 and January 2025 were initially assessed for eligibility. At our institution, coronary angiography is performed according to the contemporary guideline-directed indications for suspected or known coronary artery disease in a stable setting. The indications include abnormal or high-risk non-invasive testing suggestive of myocardial ischemia (e.g., stress ECG, stress echocardiography, or myocardial perfusion imaging); persistent or progressive angina symptoms despite optimal medical therapy; evaluation of equivocal or discordant non-invasive test results in patients with intermediate–high pre-test probability; and pre-procedural assessment in selected patients with structural heart disease when concomitant CAD is suspected [9]. Patients presenting with acute coronary syndrome (n = 124), previous myocardial infarction (n = 25), a history of cardiac surgery (n = 35), atrial fibrillation (n = 24), bundle branch block (n = 17), pacemaker rhythm (n = 4), second or third degree atrioventricular block (n = 4), or missing data (n = 7) were excluded. After applying these criteria, the final study cohort consisted of 314 patients (Figure 1). Patient demographics and laboratory parameters were obtained from hospital medical records. The study was approved by the institutional review board and adhered to the STROBE guidelines.

### 2.2. Electrocardiography

A twelve-lead surface ECG that was taken prior to coronary angiography was evaluated. The electrical risk score was obtained from the ECGs. The electrical risk score consists of 6 parameters: heart rate (>75 bpm); left ventricular hypertrophy (Sokolow–Lyon criteria); delayed QRS transition area (≥V5), frontal QRS-T angle (>90°), long QTc interval (>450 ms for men and >460 ms for women); and long T peak to T end interval (Tp-e) (>89 ms).

Each parameter was scored as 1 if it exceeded the specified threshold (abnormal) and 0 if it was within normal limits. The sum of these six binary parameters constituted the ERS for each patient, resulting in a total score ranging from 0 (no abnormalities) to 6 (all criteria met). This composite score provides a quantitative assessment of electrical instability based on the established ECG markers [5].

### 2.3. Coronary Angiography

Coronary angiographies were performed via radial and/or femoral access using standard Judkins catheters. Patients with ≥70% stenosis in one or more major epicardial arteries or ≥50% in the left main coronary artery were included in the coronary artery disease group, and patients with no plaques, stenosis, or myocardial bridge were defined as the control group.

### 2.4. Statistical Analysis

Continuous variables were presented as either mean ± standard deviation or median (interquartile range), depending on their distribution, while categorical variables were expressed as frequencies and percentages. The normality of continuous data was assessed using the Shapiro–Wilk test, and the homogeneity of variances across groups was evaluated by Levene’s test. For comparisons involving categorical variables, the Chi-square test was applied. Parametric tests (independent samples *t*-test) were used for normally distributed continuous variables, whereas non-parametric variables were analyzed using the Mann–Whitney U test. Additionally, multivariate logistic regression analysis was performed to determine independent predictors of coronary artery disease. Correlation analysis between continuous variables was conducted using Pearson’s correlation analysis.

The statistical analysis was conducted via IBM SPSS Statistics Version 20.0 software package.

## 3. Results

The mean age of the study population was 57.8 ± 11.4, and 61.5% (n = 193) were male. The most common comorbidities among the whole population were hypertension (45.2%), diabetes mellitus (30.6%), and smoking (49.7%). Electrical risk score was 0 for 9.9% (n = 31), 1 for 25.2% (n = 79), 2 for 31.8% (n = 100), 3 for 20.7% (n = 65), 4 for 7.6% (n = 24), 5 for 4.5% (n = 14), and 6 for 0.3% (n = 1) of the patients.

The study population was assessed with two groups. A total of 158 patients were included in the coronary artery disease group, and 156 were included in the control group. The coronary artery disease group has more patients with a history of hypertension and smoking, but other than that, the study groups were similar in terms of comorbidities. The demographic properties and laboratory results are demonstrated in Table 1.

The mean ERS of the study population was 2.06 ± 1.27. The mean ERS was 2.34 ± 1.35 for the CAD group and 1.78 ± 1.12 for the control group. The study groups were analyzed, and the difference in ERS among the groups was statistically significant (*p* = 0.006) (Table 2). Individual ERS parameters did not show any statistical difference between the groups in terms of heart rate, left ventricular hypertrophy, or wide frontal QRS-T angle (*p* > 0.05). The coronary artery disease group had more abnormalities for delayed QRS transition zone (*p* = 0.023), prolonged QTc (*p* = 0.004), and prolonged T peak to end interval (*p* = 0.001) (Table 3).

**Figure 2:**
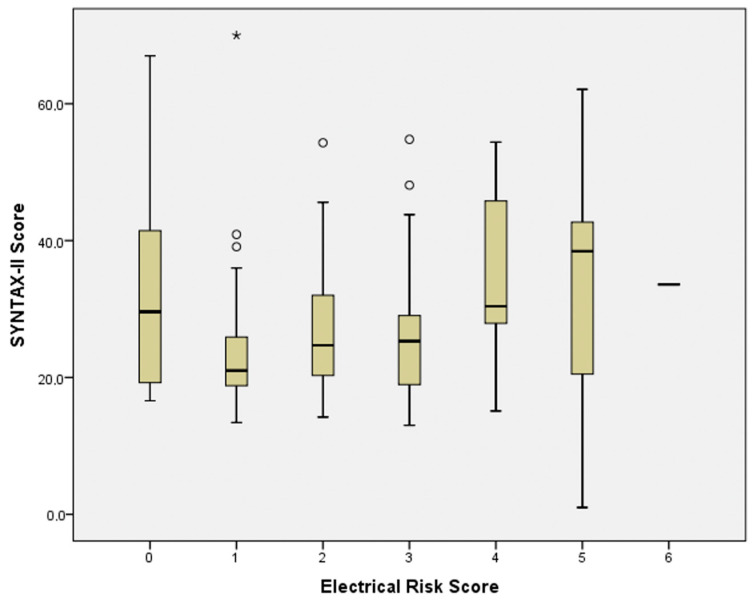
The boxplot graph of ERS and SYNTAX-II scores. (Asterisk represents the range of values within 1.5 times the interquartile range (IQR) from the lower and upper quartiles. Circles indicate mild outliers beyond this range).

By analyzing the coronary artery disease group within itself, the median SYNTAX-II score of the group was found to be 25.2 (IQR = 19.5–34.7). When correlation analysis was performed for the ERS and SYNTAX-II scores, the relationship was found to be statistically significant (r = 0.173, *p* = 0.033). Figure 2 shows the boxplot graph of the ERS and SYNTAX-II scores.

When the multivariate logistic regression analysis was performed, the electrical risk score (ERS) emerged as an independent predictor of coronary artery disease (odds ratio [OR] = 1.338, 95% confidence interval [CI]: 1.036–1.728, *p* = 0.026), along with age (OR = 1.076, 95% CI: 1.040–1.114, *p* < 0.001), gender (OR = 0.473, 95% CI: 0.226–0.991, *p* = 0.047), hypertension (OR = 2.159, 95% CI: 1.083–4.302, *p* = 0.029), high-density lipoprotein (HDL) cholesterol (OR = 0.943, 95% CI: 0.911–0.976, *p* = 0.001), low-density lipoprotein (LDL) cholesterol (OR = 1.011, 95% CI: 1.002–1.020, *p* = 0.012), leukocyte count (OR = 1.156, 95% CI: 1.032–1.295, *p* = 0.012), and ejection fraction (OR = 0.909, 95% CI: 0.863–0.957, *p* < 0.001). These results indicate that in addition to conventional risk factors, a higher ERS significantly increases the likelihood of angiographically confirmed coronary artery disease, supporting its potential use in clinical risk stratification (Table 4).

## 4. Discussion

The main findings of this study are that there is a significant relationship between ERS and the severity of coronary artery disease (CAD), and that ERS is a parameter that can be used to predict CAD. In stable patients who underwent coronary angiography, higher ERS was clearly linked to having CAD and to more severe disease. This association stayed significant even after accounting for common risk factors, suggesting ERS adds useful information beyond routine ECG ischemia signs. Among ERS components, delayed precordial QRS transition, prolonged QTc, and prolonged Tp-e interval were the most abnormal in the CAD patients. These findings point to activation delay and repolarization heterogeneity as key electrical features connected with anatomical CAD, and support using these measures—within ERS and possibly on their own—for non-invasive risk stratification.

ECG is undoubtedly the first test to be performed in case of suspected cardiac disease [10]. The electrical risk score is a scoring system that combines many ECG parameters. This scoring system was defined by Aro et al. and consists of six parameters [5]. This scoring system was found to be related to conditions such as sudden cardiac death and acute coronary syndrome [6,7]. When patients with acute coronary syndrome were examined, it was found that cardiac risk and in-hospital mortality were higher in patients with high ERS [6]. Our findings align with previous research, such as the study by Aro et al. [5], which demonstrated that a cumulative electrical risk score (ERS) derived from six ECG parameters correlates with an increased risk of arrhythmias and sudden cardiac death. Distinct from earlier studies that focused primarily on arrhythmic risk in general populations, our study targeted patients with suspected stable coronary artery disease undergoing coronary angiography. Importantly, we found that abnormalities specifically in the QRS transition zone, prolonged QT intervals, and T peak to T end intervals were more frequent in patients with angiographically confirmed CAD. These results are consistent with the previous literature, indicating that ERS parameters can predict worse coronary artery outcomes in at-risk patients. Thus, our work extends the utility of ERS beyond arrhythmic risk to the identification and stratification of CAD in a clinically relevant cohort.

Elmas et al. first showed that ERS is impaired in hypertensive patients with the non-dipper blood pressure pattern—an established risk factor for CAD [11]. In our stable angiography cohort, ERS was likewise elevated, with notable abnormalities in delayed QRS transition, prolonged QTc, and prolonged Tp-e. Taken together, these findings suggest that ERS may reflect shared pathophysiological features across non-dipping hypertension and stable CAD, indicating potential clinical relevance as a simple, non-invasive ECG marker that can complement conventional assessment.

Disruptions in the QTc interval and T peak to T end intervals are markers for ventricular repolarization delay and may show ventricular arrhythmia tendency [5,12,13]. Prolongation in these parameters shows prolongation in action potential duration, ventricular damage, and problems in ionic channel controls [8,14]. These parameters exhibit deterioration in myocardial ischemia and heart failure, and our study demonstrates that they are statistically significantly impaired in patients with coronary artery disease (CAD) [8,15]. Notably, acute coronary syndrome was excluded from the patient cohort, highlighting that even in the absence of unstable angina or positive cardiac marker values, these parameters can remain compromised in individuals with CAD, potentially predicting deterioration in myocardial repolarization. Thus, our findings indicate that these abnormalities warrant independent assessment, separate from other clinical presentations and ECG characteristics. Such alterations should heighten clinical suspicion for coronary artery disease, serving as critical indicators of significant coronary pathology in patients where CAD is suspected.

The QRS transition zone is an electrocardiographic parameter defined by the precordial lead where the R wave amplitude equals or exceeds that of the S wave. Recent studies have linked delayed QRS transition with increased mortality and higher risk of sudden cardiac death [7,16,17]. The findings from our study demonstrate that patients diagnosed with coronary artery disease exhibited a significantly delayed QRS transition zone when compared to the control group. Specifically, we observed a delayed QRS transition zone in 48% of the patients with confirmed coronary artery disease. This parameter, which constitutes a component of the electrical risk score, not only serves as part of the scoring system for the identification of coronary artery disease, but our results also suggest that it may be utilized as an independent marker for the detection of this pathology.

Building on the overall findings, well-established clinical determinants—age, sex, and hypertension—remained independent predictors of coronary artery disease (CAD) [9,18]. Beyond these conventional factors, our analysis highlighted a previously underexplored observation: patients with CAD exhibited a higher electrical risk score (ERS) despite the exclusion of acute coronary syndrome. This elevation was evident in both group comparisons and logistic regression, indicating that ECG-derived metrics not routinely used for ischemia detection may still carry diagnostic value for CAD. ERS aggregates six electrocardiographic markers, each reflecting electrical or structural myocardial damage [5,19,20,21]. Within this framework, non-invasive ECG variables plausibly act as early indicators tied to autonomic imbalance (heart rate, QTc, and Tp-e), repolarization abnormalities (QTc, QRS angle, and Tp-e), and cardiac hypertrophy (QTc, QRS angle, QRS transition, and Tp-e)—all recognized correlates of adverse outcomes in chronic heart failure and CAD [6,7,22].

In parallel, our data provide complementary evidence regarding the specific contribution of ERS and its subcomponents. We observed a significantly elevated ERS among the angiographically confirmed CAD cases, a finding that persisted after excluding acute presentations. This pattern emerged consistently across unadjusted comparisons and multivariable models, suggesting that the selected ERS elements—particularly delayed precordial QRS transition, prolonged QTc, and prolonged Tp-e—may offer clinically useful signals beyond overt ischemic ECG changes. Rather than supplanting established predictors, these parameters appear to enrich the electrocardiographic characterization of suspected CAD and warrant consideration as adjunctive markers pending validation in prospective, multicenter cohorts.

The ERS is a composite score derived from six distinct electrocardiographic markers, each of which reflects various aspects of cardiac electrical and structural damage. In this context, it is plausible that these non-invasive ECG variables may serve as earlier indicators of mortality risk, as they depend on imbalanced autonomic control (heart rate, QTc, and Tp-e), repolarization alterations (QTc, QRS-angle, and Tp-e), and cardiac hypertrophy (QTc, QRS-angle, QRS transition, and Tp-e), all of which are well-established prognostic markers for cardiac disorders [6,7,8,16]. This study emphasizes once again the need to give greater importance to these parameters.

## 5. Limitations

The present study has several limitations that should be acknowledged. First, it is based on a single-center design, which restricts the generalizability of the findings to broader and more diverse patient populations. Multicenter studies are, therefore, recommended to validate the reproducibility of these results. Second, the study relied on retrospective data collection, which may introduce potential biases and incomplete information; future prospective studies would provide more complete and reliable data. Third, the electrical risk score (ERS) used in this study has not yet been validated against anatomical parameters, such as specific coronary angiographic lesions or physiological assessments obtained from echocardiographic evaluations. Such validation would be essential to confirm the pathophysiological relevance and clinical robustness of the ERS. Finally, the lack of long-term follow-up limits the ability to assess the prognostic value of ERS in predicting cardiovascular events and mortality. To address these issues, a large-scale, multi-institutional, prospective cohort study with a more comprehensive statistical approach is warranted.

## 6. Conclusions

In conclusion, this study elucidates the significant association between the ERS and the presence and severe CAD in stable patients. Our findings indicate that the ERS, with its integration of various electrocardiographic parameters, could serve as a valuable non-invasive tool for risk stratification in CAD.

## Figures and Tables

**Table 1:** The demographic properties and laboratory results of the patient groups.

	Control Group (n = 156)	Coronary Artery Disease Group (n = 158)	*p*
Gender n,%	80 (51.3%)	113 (71.5%)	<0.001
Hypertension n,%	84 (53.8%)	58 (36.7%)	0.002
Diabetes mellitus n,%	48 (30.8%)	48 (30.4%)	0.940
Peripheral artery disease n,%	3 (1.9%)	2 (1.3%)	0.642
Chronic obstructive pulmonary disease n,%	8 (5.1%)	6 (3.8%)	0.568
Stroke n,%	0 (0%)	2 (1.3%)	0.159
Chronic kidney failure n,%	1 (0.6%)	6 (3.8%)	0.058
Malignancy n,%	1 (0.6%)	1 (0.6%)	0.993
Smoking n,%	65 (41.7%)	91 (57.6%)	0.005
Creatinine, mg/dL	0.8 (0.7–0.97)	0.9 (0.8–1.08)	<0.001
Glomerular filtration rate	90.3 ± 19.1	79.6 ± 22.0	<0.001
Total Cholesterol, mg/dL	197.2 ± 38.3	193.1 ± 49.2	0.441
High-density lipoprotein cholesterol, mg/dL	47.8 ± 12.2	40.9 ± 9.3	<0.001
Low-density lipoprotein cholesterol, mg/dL	118.2 ± 32.8	121.6 ± 40.9	0.430
Triglyceride, mg/dL	137.5 (90–202)	128 (87.5–193)	0.303
Hemoglobin g/dL	13.8 ± 1.7	13.9 ± 2.1	0.359
Leucocyte count, ×10^3^/μL	7.8 (6.6–9.3	9.7 (7.4–12.1)	<0.001
Platelet count, ×10^3^/μL	230.5 (199–269.2)	237 (191–285.5)	0.503
Ejection fraction, %	60 (58–62)	55 (50–60)	<0.001

**Table 2:** Numerical display of electrical risk score results between 0 and 6 in groups and comparison of ERS results between groups.

Electrical Risk Score	Control Group (n = 156)	Coronary Artery Disease Group (n = 158)	*p*
0	20	11	0.006
1	46	33	
2	51	49	
3	28	37	
4	10	14	
5	1	13	
6	0	1	

**Table 3:** Comparison of the parameters constituting the electrical risk score between the groups.

Electrical Risk Score	Control Group (n = 156)	Coronary Artery Disease Group (n = 158)	*p*
Heart rate n,%	76 (48.7%)	89 (56.3%)	0.177
Left ventricle hypertrophy n,%	32 (20.5%)	23 (14.6%)	0.165
Delayed QRS transition zone n,%	55 (35.3%)	76 (48.1%)	0.023
Wide frontal QRS T angle n,%	38 (24.3%)	51 (32.3%)	0.119
Prolonged QTc n,%	28 (17.9%)	51 (32.3%)	0.004
Prolonged T peak to end interval n,%	48 (30.8%)	79 (50.0%)	0.001

**Table 4:** Independent predictors of coronary artery disease according to multivariate logistic regression analysis.

	Odds Ratio	95% Confidence Interval	*p*
Electrical risk score	1.338	1.036–1.728	0.026
Age	1.076	1.040–1.114	<0.001
Gender	0.473	0.226–0.991	0.047
Hypertension	2.159	1.083–4.302	0.029
Diabetes Mellitus	0.735	0.366–1.475	0.386
Peripheral Artery Disease	0.624	0.051–7.671	0.712
Chronic Renal Failure	1.460	0.1.4–20.425	0.779
Malignancy	3.006	0.035–260.225	0.629
Smoking	0.518	0.246–1.093	0.084
Creatinine	0.976	0.608–1.566	0.919
High-density lipoprotein cholesterol	0.943	0.911–0.976	0.001
Low-density lipoprotein cholesterol	1.011	1.002–1.020	0.012
Triglyceride	0.997	0.993–1.000	0.051
Hemoglobin	0.873	0.722–1.056	0.163
Leukocyte count	1.156	1.032–1.295	0.012
Platelet count	1.003	0.998–1.007	0.279
Ejection fraction	0.909	0.863–0.957	<0.001

## Data Availability

The data supporting the findings of this study are not publicly available but can be obtained from the corresponding author upon reasonable request. Since institutional permission is required for the use of the data, it cannot be shared as publicly available.
